# 肺部亚厘米结节诊疗进展

**DOI:** 10.3779/j.issn.1009-3419.2020.102.11

**Published:** 2020-05-20

**Authors:** 依侣 吕, 波 叶

**Affiliations:** 200030 上海，上海交通大学附属胸科医院胸外科 Department of Thoracic Surgery, Shanghai Chest Hospital, Shanghai Jiao Tong University, Shanghai 200030, China

**Keywords:** 肺部亚厘米结节, 诊断, 治疗, Subcentimeter pulmonary nodules, Diagnosis, Treatment

## Abstract

随着高分辨多层螺旋计算机断层扫描(computed tomography, CT)的广泛应用和定期体检的普及，临床诊断的肺部亚厘米结节的数量日益增多。肺部亚厘米结节的恶性概率和恶性程度较低，诊治难度较高，易误诊、漏诊，因此肺部亚厘米结节的定性和诊疗策略一直为临床工作的重点难点和研究热点。本文对肺部亚厘米结节病变的性质评估、处理治疗及预后等方面的进展进行了综述，并对肺部亚厘米结节的临床诊疗进行了总结及分析。

## Abstract (en)

With the widespread use of high-resolution multislice spiral computed tomography and the popularization of regular physical examinations, the prevalence of clinically diagnosed subcentimeter pulmonary nodules is increasing. Subcentimeter pulmonary nodules have low malignant probability, however, the diagnosis and management are of high difficulty and it is likely to misdiagnose and miss malignant nodules. Therefore, the evaluation and management of subcentimeter pulmonary nodules have always been the key points of clinical work. This article reviews and summarizes the progress in the evaluation and management of subcentimeter pulmonary nodules.

肺癌是全球最常见的癌症死亡原因^[1]^。自本世纪初以来，肺癌已成为我国发生率和死亡率最高的癌症，2015年中国国家癌症中心的统计研究^[2]^显示肺癌的5年生存率仅有16.1%，这一数据远低于其他恶性肿瘤。近年来随着高分辨多层螺旋计算机断层扫描(computed tomography, CT)的广泛应用，不仅早期筛查出的肺部结节数量增多，而且结节的直径也越来越小。肺部亚厘米结节的定性和诊疗策略一直为临床工作的重点难点和研究热点，本文对肺部亚厘米结节病变的性质评估、处理治疗及预后等方面的进展进行了综述，并对肺部亚厘米结节的临床诊疗进行了总结及分析。

## 1 肺部亚厘米结节的性质

近年来一个类别的肺部结节日益增多即肺部亚厘米结节，目前美国胸科医师学会最新发布的第三版肺癌的诊断和治疗指南中将肺部亚厘米结节定义为直径≤ 8 mm的肺结节^[3]^。

依据结节密度分类，肺部亚厘米结节可分为纯磨玻璃结节、部分磨玻璃结节和纯实性结节。磨玻璃结节是局限性的肺内磨玻璃密度影，肺内磨玻璃密度影是由气腔部分充盈、肺间质增厚、肺泡的部分塌陷、肺毛细血管血容量的增加等多种因素综合导致的，CT表现为密度轻度增高但其内血管及支气管轮廓尚可见，实性结节表现为均匀的软组织密度且其内血管和支气管影像被掩盖，部分磨玻璃结节介于两者之间^[4]^。

依据结节的良恶性可将肺部亚厘米结节分为良性结节和恶性结节。病理组织学结果为判断结节良恶性的金标准，恶性肺部亚厘米结节的病理结果多为肺腺癌。根据2011年国际肺癌研究协会(International Association for the Study of Lung Cancer, IASLC)/美国胸科学会(American Thoracic Society, ATS)/欧洲呼吸学会(European Respiratory Society, ERS)的肺腺癌分类，肺腺癌的病理分类可分为非典型腺瘤性增生，原位癌，微小浸润性腺癌，浸润性腺癌和浸润性腺癌变异型^[5]^，其中非典型腺瘤性增生在影像上多为纯磨玻璃结节^[6]^。8项较大的荟萃分析^[7]^结果显示肺部恶性结节的发生率从1.1%到12%不等，且结节的恶性概率与直径有关，肺部亚厘米结节的恶性程度整体较低。

## 2 肺部亚厘米结节的良恶性可能性评估

在临床工作中，结节的良恶性评估是诊治的重点和难点，影响了后续治疗方案的制定和疾病预后等各个方面，而结节良恶性评估应结合患者的临床信息和影像学特征进行综合考虑。

### 2.1 临床评估

年龄、吸烟和恶性肿瘤史为肺癌的三大独立危险因素^[8]^。其中年龄是最重要的危险因素之一，30岁以下患肺癌死亡的患者不到1%，超过40岁的肺癌患者死亡率陡然上升，超过70岁的患者肺部结节有88%的可能性为肺癌^[9]^，然而有研究^[10]^显示感染免疫缺陷病毒的年轻患者易患肺癌或其他癌症。吸烟是另一独立危险因素，且危险程度和吸食香烟的量和时长呈正比^[11]^。有恶性肿瘤或曾患有恶性肿瘤的患者发生肺癌的可能性上升，值得注意的是，有研究^[12]^表明在患有黑色素瘤、肉瘤或睾丸癌的患者中，恶性结节为转移的可能性是原发性的2.5倍，而在患有头颈部鳞癌的患者中，肺部恶性结节为转移的可能性是原发性的8倍。其他危险因素包括肺纤维化、慢性阻塞性肺疾病(chronic obstructive pulmonary disease, COPD)病史、氡气、石棉等^[13]^。

### 2.2 影像学特征

判别恶性结节最主要的影像学特征首先是其直径。多项研究^[14-17]^表明，肺部结节的直径越大，结节为恶性的可能性也越大。Wahidi等^[7]^对过去的文献进行了整理总结，其研究表明，结节直径与恶性程度密切相关，结节直径 < 5 mm的恶性率为0%-1%，5 mm-10 mm的恶性率为6%-28%，直径 > 20 mm的结节恶性率为64%-82%。Horeweg等^[18]^对NESLON肺癌筛查试验的数据进行了分析研究，结果显示直径 < 5 mm的肺部结节的恶性概率为0.4%，而直径为5 mm-10 mm的肺部结节恶性概率上升至1.3%，直径 > 10 mm的肺部结节的恶性概率为15.2%。这两项研究的结果表明肺部直径 < 5 mm的结节恶性概率 < 1%，Wahidi等^[7]^的研究是综合了之前的文献因此结果跨度较大，从大型筛查试验的结果可以看出直径 > 5 mm的肺部亚厘米结节的恶性率也相对较低。

其次，结节的质地对于判断结节良恶性也很重要，CT表现为部分实性结节和纯磨玻璃结节的恶性率高于实性结节。有研究^[7]^表明纯磨玻璃样结节的恶性率为59%-73%，相较于纯实性结节的恶性率为7%-9%，磨玻璃结节为恶性结节的可能性较高。Henschke等^[19]^的研究显示，实性结节最为常见(56%)，其次为纯磨玻璃结节(12%)和部分实性结节(7%)，恶性率最高的为部分实性结节(63%)，其次为纯磨玻璃结节(18%)和实性结节(7%)。

再次，结节的位置与恶性程度也密切相关，有研究表明恶性结节多分布于右肺和上叶，有近70%的恶性结节位于肺上叶，良性结节可分布于全肺，有学者推测这一特性可能和吸烟导致的致癌物质堆积于肺的上部有关，值得注意的是结节的位置并非恶性程度的独立风险因素^[20, 21]^。此外，大约近一半的肺腺癌表现为外周的孤立结节，而表现为孤立性肺结节的鳞状细胞癌更可能发生于中央。

最后，多项研究^[15, 22, 23]^表明不规则、毛刺、分叶状的边缘与恶性结节有关，但这些边缘特征并非为诊断性的，良性结节也有可能出现此种边缘特征，此外，有研究表明21%的恶性结节也有光滑规则的边缘^[24]^。

除了上述影响因素之外，结节的钙化、脂肪化和肺裂周围结节通常提示结节性质为良性。

## 3 肺部亚厘米结节的处理

肺部亚厘米结节的处理主要包括：① CT观察随访；②进一步的诊断性检查；③临床治疗。对于低风险的结节应进行CT观察随访，对于高风险的结节可根据情况选择内外科治疗，处于两者之间的结节可行进一步的诊断性检查。

### 3.1 随访观察

多项大型筛查试验表明可以对恶性概率较低的低风险人群进行定期的CT随访观察。2015年的一项筛查试验显示，在2, 392例纯磨玻璃结节患者中，有26% (628/2, 392)的患者结节变小或消失。由于大多数的恶性结节的倍增时间为20 d-400 d，因此临床上倾向于超过2年的稳定结节为良性结节^[25]^。随访观察的缺点在于结节可能在随访间期内发生恶变或进展甚至转移，因此应谨慎评估结节并选择合适的观察间期。通过随访观察确认的生长加速或实性成分的增加将需要进一步的评估。

近年来有不少国外学者尝试使用磁共振成像(magnetic resonance imaging, MRI)代替CT进行肺部结节的筛查和随访，旨在降低医疗辐射导致的患癌风险。已有的多项研究显示，使用MRI检测直径为4 mm-8 mm的小结节的灵敏度可达到60%-90%，而检测直径 > 8 mm的结节的灵敏度可达到100%，但即使是在最佳条件下，使用三维梯度回声核磁共振或T2加权快速自旋回声核磁共振能检测到的肺结节直径最小在3 mm-4 mm左右^[26-29]^。与实性结节相比，MRI检测磨玻璃结节的相关研究相对较少，Meier-Schroers等^[29]^对223例受试者同时使用MRI和低剂量螺旋计算机断层扫描(low-dose computed tomography, LDCT)进行了长达5年的筛查试验，研究结果显示直径为4 mm-5 mm的实性结节的诊断率为69.3% (61/88)，直径 > 6 mm的实性结节诊断率为97.4% (37/38)，而直径 < 20 mm的磨玻璃结节诊断率为72.7% (8/11)，三个未检测出的结节直径分别为4 mm、5 mm和7 mm，均为亚厘米结节。由于肺部亚厘米结节恶性概率低，针对可疑结节可先进行随访观察，但随访观察的时间较长，若能使用MRI代替CT进行随访检查可大大降低医疗辐射带来的风险，但现有的研究表明针对肺部亚厘米结节MRI尚不能代替CT检查。

### 3.2 诊断性检查

对于难以明确性质的患者可选用其他诊断性检查如CT引导下细针穿刺活检(fine needle aspiration biopsy, FNAB)和支气管镜引导的活检等。CT引导下FNAB是临床常用的肺活检方法，尤其适用于靠近胸壁的结节或病变。以往的研究表明与大直径的结节相比其准确性降低且并发症发生率上升^[30]^。另外由于肺部磨玻璃结节穿刺活检的特异性较差易误检，国内指南并不提倡小直径的肺部磨玻璃结节进行术前活检^[31, 32]^。支气管镜引导的活检主要包括支气管刷细胞学(bronchial brush cytology, BB)、支气管肺泡灌洗(bronchial alveolar lavage, BAL)和经支气管肺活检(transbronchial lung biopsy, TBLB)。近年来有学者将电磁导航支气管镜和支气管内超声相结合用于引导经支气管肺活检，提高了肺部亚厘米结节诊断的灵敏性^[33, 34]^。

### 3.3 临床治疗

随着微创外科的发展，电视胸腔镜手术由于其创伤小、死亡率低、患者恢复快、住院时间短等优势已成为肺部结节的最佳治疗方案。临床诊断为恶性或可疑恶性的结节应接受外科手术切除治疗，肺叶切除术及纵隔淋巴结清扫为早期肺癌的标准手术方案，其优点在于这种手术方式可以完整的切除整个肿瘤并且去除外周和中央的淋巴引流通路，减少复发风险^[35]^。2002年Miller等^[36]^的研究表明，淋巴结转移存在于 < 1 cm的肺部结节中，接受肺叶切除术的患者相比接受楔形切除术和肺段切除术的患者有着更好的生存率和更低的复发率，因此肺叶切除术和纵隔淋巴结清扫一直以来为首选手术方案。近年来，由于肺段切除术和楔形切除术具有死亡率低、创伤小、保留肺功能等优点，显示为磨玻璃性质的肺部亚厘米结节逐渐开始选用肺段切除或者楔形切除的方式进行切除。Kato等^[37]^建议直径 < 2 cm且GGO占比达到80%以上可选用局部切除术代替肺叶切除术。由于多项研究显示对于早期肺癌患者来说肺叶切除术和肺段切除术以及楔形切除术在术后生存率上并无统计学差异，因此在术中区域淋巴结未转移的情况下，局部切除可取代肺叶切除术^[38-41]^。

除了微创外科治疗，其他微创治疗方式如消融、放射粒子植入、不可逆电穿孔等也可用于无法外科切除的病灶或患者无法耐受外科手术的情况。消融技术包括传统的立体定向放射治疗和经皮穿刺消融技术如射频消融、微波消融和冷冻消融等。立体定向放射治疗(stereotactic ablative radiotherapy, SABR)是无法手术的早期非小细胞肺癌患者首选的治疗方式，2017年Sun等^[42]^发布了一项Ⅱ期前瞻性试验的7年随访研究结果，是目前关于SABR的前瞻性临床试验中随访时间最长的临床研究，其结果显示使用SABR治疗的早期肺癌患者的总体生存率和局部控制率不逊于手术治疗的结果。其他的微创介入方式多为单中心、小样本的研究，且较少有关于应用于肺部亚厘米结节的研究，但微创治疗是未来发展的方向之一，需要进一步开展研究工作使得该治疗方法得以普及和规范化应用。

### 3.4 国内外指南推荐的临床路径

2017年费莱舍尔学会发布的指南推荐，单个直径 < 6 mm的实性结节，低风险组无需进一步处理，高风险组可在12个月后进行CT复查；直径6 mm-8 mm的单个实性结节可在6个月-12个月后进行复查，进行监测；单个直径 > 8 mm的实性结节建议3个月后复查CT，正电子发射计算机断层显像(positron emission tomography/CT, PET/CT)或组织活检。单个直径 < 6 mm的磨玻璃结节或部分实性结节无需特殊处理，单个直径 > 6 mm的磨玻璃结节建议在6个月-12个月时复查CT，并在之后5年每2年复查一次CT。建议的随访间隔旨在最大限度地减少检查次数和诊断前CT随访期间癌症进展的可能性^[17]^。美国胸科医师协会在2007年发布的第二版指南指出有肺部亚厘米结节的患者复查CT的频率和间隔应综合多方面考虑，包括临床的危险因素、结节的大小、形状、结节的生长速率等，并且由于临床上并无证据显示早期发现肺部亚厘米结节可改善其死亡率，推荐采用侵略性相对较低的方法对其进行处理^[43]^。在2013年美国胸科医师协会发布的最新的指南中，考虑到肺部亚厘米结节的恶性率较低，肺部亚厘米结节的处理仍以CT观察随访为主，在随访期间直径增大和纯磨玻璃质地中出现实性成分的结节应进行进一步评估或视患者情况考虑直接外科切除^[3]^。我国最新发布的2018年版肺部结节诊治中国专家共识中推荐无肺癌危险因素的患者根据结节大小≤ 4 mm、4 mm-6 mm、6 mm-8 mm分别采取选择性采访、12个月后影像学随访(如无变化则之后年度随访)、6个月-12个月后随访(如无变化则18个月-24个月后再度随访，其后转为常规年度检查)。纯磨玻璃结节以5 mm为界观察，直径≤ 5 mm在6个月随访，直径 > 5 mm在3个月随访观察，如结节直径增长(尤其是 > 10 mm)或出现实性成分增加则需考虑非手术活检(直径 > 10 mm)或手术切除。单个直径≤ 8 mm的部分磨玻璃结节推荐在3个月、6个月、12个月、24个月进行随访，如无变化转为常规年度检查，如实性成分增加则考虑手术切除。我国发布的2018版肺部结节诊治指南对肺部亚厘米结节推荐措施也以随访为主，如随访过程中出现直径增长或实性成分增加则根据实性结节和亚实性结节的不同分别采取下一步措施^[44]^。

## 4 肺部亚厘米结节的预后

肺部亚厘米良性结节的预后良好，恶性结节的预后与多种因素相关。多项研究^[36, 45-47]^显示外科手术后的5年生存率为82%-86%，复发率为9%-18%。此外，结节的质地与预后密切相关。Hattori等^[48]^对328例进行手术切除的肺部亚厘米结节患者进行分析，结果表明纯磨玻璃结节的5年生存率和无复发生存率明显优于实性结节和部分实性结节。纯磨玻璃结节的5年生存率和无复发生存率均为100%，部分实性结节为97.5%和94.9%，纯实性结节为87.6%和79.3% (*P* < 0.000, 1)。Sakurai等^[49]^对291例有肺部亚厘米结节且手术切除后病理显示为恶性的患者进行了研究，结果显示所有的纯磨玻璃结节和部分磨玻璃结节均无淋巴结转移，10% (9/90)的实性结节患者发生了淋巴结转移，纯磨玻璃结节和磨玻璃成分 > 50%的部分磨玻璃结节的患者的5年生存率为100%，磨玻璃成分 < 50%的部分磨玻璃结节患者的5年生存率为98%，实性结节患者的5年生存率为88%。

## 5 总结

由于影像学技术的发展和大众定期体检意识的增强，越来越多的肺部亚厘米结节被发现。肺部亚厘米结节的临床诊疗应侧重于减轻患者的心理和经济负担的同时综合考虑手术带来的利弊，然后选择合适的处理方案。肺部亚厘米结节的恶性概率较低，尤其是磨玻璃结节恶性程度低、增长缓慢且预后良好，因此临床上可视情况优先选择观察随访。观察随访中出现结节直径增长或实性成分的增加应视情况考虑进一步的检查或手术切除。微创治疗一直以来都是肺癌治疗发展的方向之一，但目前关于肺部亚厘米结节的研究较少，各种治疗方式在有效性和预后等方面是否可以代替外科手术治疗有待于进一步研究。
